# The optimization study of the operational ventilation system for long tunnels on secondary highways

**DOI:** 10.1038/s41598-025-31830-4

**Published:** 2026-01-21

**Authors:** Xinjiang Wei, Shuai Li, Xiao Wang, Shiming Du, Tongchun Han

**Affiliations:** 1 Department of Civil Engineering, Hangzhou City University, Hangzhou, 310015 China; 2 China Southern Power Grid Energy Storage Co.,Ltd., Guangzhou, 510000 China; 3 Ningbo Shuntong Group Co., LTD, Ningbo, 315400 China; 4 College of Civil Engineering and Architecture, Zhejiang University, Hangzhou, China

**Keywords:** Long highway tunnels, Combined ventilation, Tunnel operation, Air quality, Traffic congestion, Environmental sciences, Engineering

## Abstract

Long tunnels and extremely long tunnels on highways are becoming increasingly common. As tunnel lengths continue to increase, pollutants in the air can easily accumulate inside the tunnels, leading to air quality that fails to meet driving requirements, which poses health risks to drivers and passengers. This paper uses the Donggang Mountain secondary highway long tunnel as the engineering background and establishes a 1:1 full-scale model using the numerical simulation software Fluent. It studies the extreme traffic congestion conditions in long highway tunnels, exploring the distribution patterns of pollutant gas concentrations within the tunnel and the role of the longitudinal ventilation system under different operating conditions in reducing tunnel pollutant gas concentrations and improving air quality. Furthermore, the study investigates the ventilation efficiency of a combined ventilation method using ventilation shafts and jet fans. Compared to the full jet longitudinal ventilation method, the carbonic oxide (CO) concentration growth rate after the ventilation shafts decreased by 50%.

## Introduction

With the rapid development of the transportation industry in China, an increasing number of highway tunnels are being constructed, and they are becoming longer. According to statistical data^1^, by the end of 2024, the total length of highways in China reached 5.4904 million km, secondary and above highways account for 14.2% of the total highway mileage. At the same time, with the continuous increase in urbanization in China, the transportation network between urban and rural areas is developing, leading to the emergence of long tunnels on secondary highways. It is noteworthy that tunnel construction and operation can induce complex soil-structure interactions, potentially affecting the mechanical behavior and integrity of tunnel linings and ventilation structures. As global low-carbon goals are promoted^2,3,4^, the sustainability of tunnel infrastructure is crucial in long tunnel design, including the design of tunnel ventilation systems and their environmental impact. During the operation of these long tunnels, the accumulation of gaseous pollutants poses a significant threat to the health and safety of personnel inside the tunnel^4,5,6^.

Due to the narrow and confined structure of highway tunnels, the internal gaseous pollutants are not easily dispersed. As traffic volume continues to increase, the concentration of gaseous pollutants inside the tunnel also rises^6–8,9^. This is particularly problematic during prolonged traffic congestion, where vehicles are idling, leading to greater emissions of pollutants. Moreover, the decreased visibility and the dispersion of hot and toxic smoke not only reduces visibility and affects safe driving but also poses health risks to individuals^10–13^. Due to the enclosed and narrow nature of tunnels, vehicle exhaust easily accumulates inside, containing a high concentration of toxic gases such as carbonic oxide (CO), nitrogen dioxide (NO_2_), and Formaldehyde (HCHO). CO is one of the most harmful gases to human health, and is known as the ‘silent killer’ (as it is odorless and colorless)^14^. After CO enters the human body, it easily combines with hemoglobin in the human blood to form carboxyhemoglobin, which reduces the ability of hemoglobin to transport oxygen. When the concentration of carboxyhemoglobin in the blood exceeds 10%, symptoms such as mild headaches and difficulty breathing occur. When the concentration exceeds 20%, symptoms such as severe headaches, nausea, and abnormal limb movements occur. When the concentration exceeds 50%, coma can occur, endangering life.

Efficient ventilation methods play a crucial role in lowering pollutant concentrations and improving air quality within tunnels^14–17^. Liu et al.^18–21^ studied carbon monoxide (CO) as a representative component of tunnel pollutants, investigating the health hazards of different CO concentrations and the distribution characteristics and trends of CO concentrations within the tunnel. Khan et al.^9,21,22^ conducted experiments and monitoring to study the concentration range and distribution characteristics of gaseous pollutants, including CO, SO_2_, and NO, in tunnels. Ling et al.^22,23^ examined the impact of natural openings and the arrangement of jet fans on the distribution of CO concentrations inside the tunnel. Liu et al.^23,24^ found that CO concentration values in the tunnel were mainly related to vehicle speed and traffic flow, showing little fluctuation with seasonal changes. Ren et al.^24,25^ used Fluent simulation software to investigate the movement and diffusion patterns of vehicle exhaust under varying vehicle spacing and speeds, concluding that CO tends to form a vortex-like distribution due to backflow from vehicle exhaust, with a slower diffusion rate at short spacing.

As tunnel lengths continue to increase, single ventilation methods often fail to meet both the ventilation demands and operational economic requirements of the tunnels. Longitudinal jet ventilation can easily lead to excessive design wind speeds inside the tunnel, while the dual-tunnel complementary ventilation method faces issues such as excessively long smoke exhaust channels. Guo et al.^25,26^ proposed a hybrid ventilation method of single-channel complementary ventilation by studying the functional characteristics of various longitudinal ventilation modes. Xia et al.^26,27^ addressed the issue of imbalanced airflow requirements between the two lanes of long highway tunnels by suggesting a dual-tunnel complementary ventilation method. Xie^27,28^ and Wang et al.^28,29^ studied the ventilation efficiency of complementary ventilation and the combination of ventilation shafts for supply and exhaust, demonstrating that shaft-based ventilation can effectively reduce the installation power of tunnel ventilation facilities. Wan^29,30^ proposed a combined ventilation method incorporating a single ventilation shaft with a cross-ventilation passage based on the Wanshui Ridge long highway tunnel, which can reduce ventilation energy consumption by 11%. Li et al.^30,31^ developed an S-shaped tunnel ventilation model that combines a top opening with jet fans, finding that when both operate simultaneously, the concentration of pollutants in the S-shaped tunnel can be reduced by 35%. Wang et al.^31,32^ studied the impact of multiple ramp separated outlets on the concentration distribution of pollutants at the exit of the Nanchang Honggu Tunnel. Zhang et al.^32,33^ validated the feasibility of natural ventilation in ultra-long highway tunnels, revealing that constructing shafts on the windward side at higher elevations and shallower depths can enhance natural ventilation, potentially reducing the need for 10 to 12 jet fans. The construction cost of the shafts can be recovered within 8 years, saving over 300,000 yuan annually in electricity costs. In addition, waterproofing measures for tunnel structures help preserve the integrity of the tunnel structure, which in turn helps control ventilation conditions within the tunnel^33,34^. The location of the vent shaft, the mechanical behavior of ventilation-shaft connecting location, as well as the construction methods of the shaft, would influence the ventilation performance^35–37,^. In total, the rational selection of tunnel ventilation schemes plays a crucial role in enhancing ventilation efficiency.

Secondary highway tunnels differ from higher-grade highway tunnels in that they cannot control the entry of non-motorized vehicles and pedestrians at their entrances and exits. Moreover, secondary highway tunnels are often single-bore, bi-directional tunnels. In the event of traffic congestion or a fire in a single-bore bi-directional tunnel, there is a risk that people upstream and downstream may become trapped. Therefore, it is essential to study the distribution patterns of pollutant gases and the operating conditions of the ventilation system under long-duration congestion in secondary highway long tunnels. Such research can better enhance ventilation efficiency and manage internal pollutant gases, providing valuable references for the design optimization of ventilation systems in long tunnels.

## Modeling the ventilation system of long tunnels on secondary highways

### Fluent numerical model verification

**Fig. 1:**
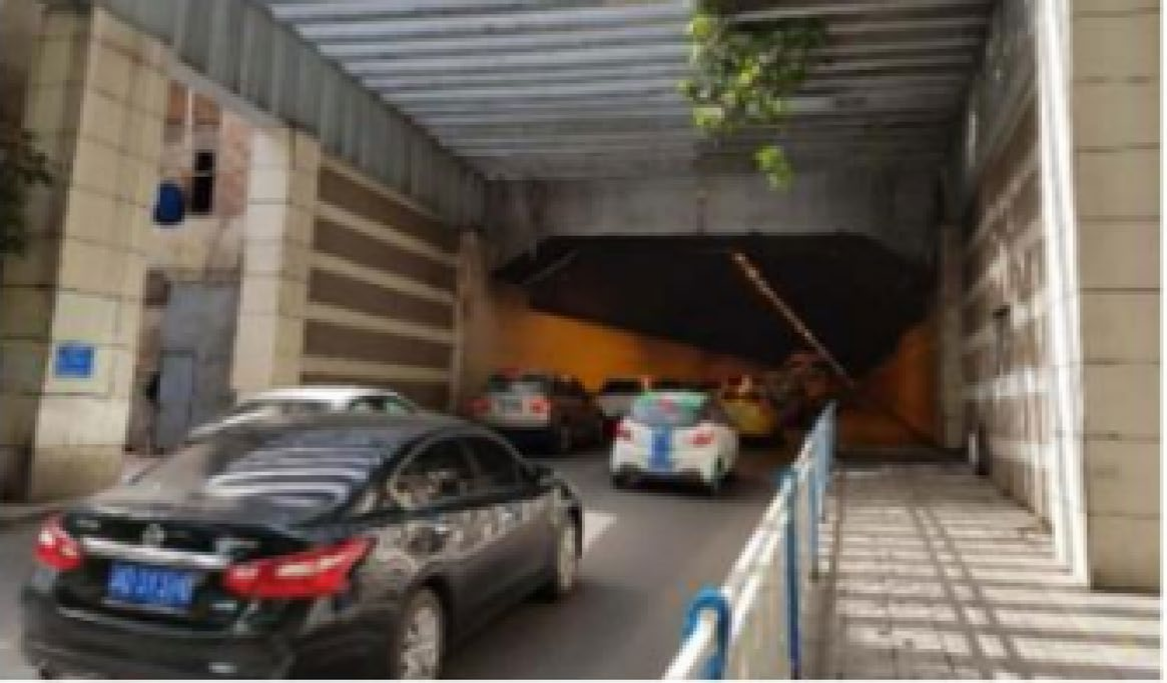
Scenic view of Bayi tunnel.

Since CO makes up a significant proportion of vehicle exhaust emissions and is highly toxic to humans, CO was selected as the characteristic pollutant for this study. To ensure the accuracy of the Fluent software simulation results, the model was validated using the Chongqing Bayi Tunnel^37,38^ as a case study, comparing the simulated pollutant concentrations with the measured data. The Chongqing Bayi Tunnel, located in the Yuzhong District of Chongqing, is an important corridor connecting Niujiatuo and Chongqing Railway Station. The tunnel experiences heavy traffic (as shown in Fig. 1), with relatively slow vehicle speeds and frequent congestion. The tunnel is 673 m long, with a clear width of 11 m and a height of 6.83 m, featuring a single-direction, dual-lane configuration. The ventilation system is natural ventilation. Data collection was conducted during the evening peak period, with measurement points chosen at distances of 100, 200, 300, 400, 500 and 600 m from the tunnel entrance. For the numerical simulation of CO concentration distribution within the tunnel, the monitoring data were collected at a height of 1.5 m from the ground, roughly corresponding to the breathing zone of a person.

**Fig. 2:**
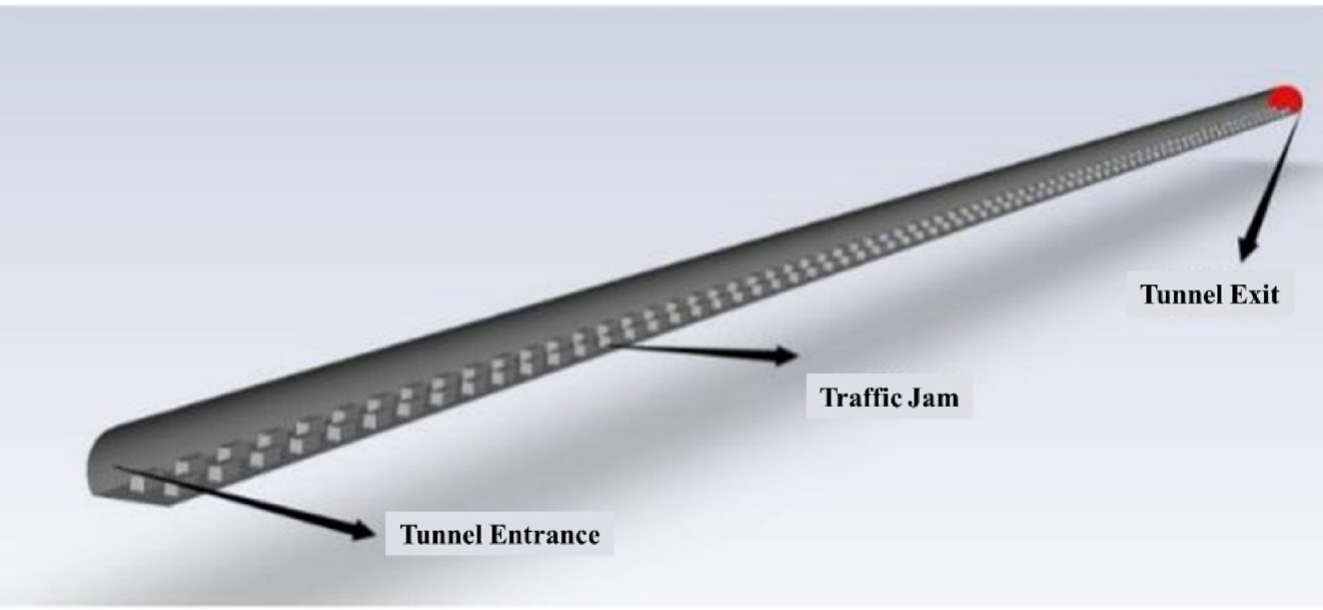
Fluent model of Bayi tunnel.

**Fig. 3:**
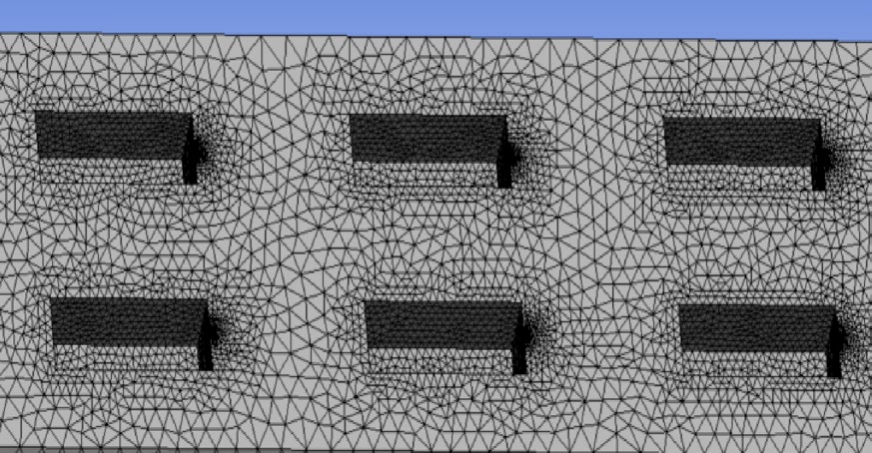
Encryption of exhaust vents and bodywork grids.

Using Fluent software, a 1:1 scale model was created to analyze the CO concentration during the operation of the Bayi Tunnel. The simulation conditions represented a traffic congestion scenario within the tunnel, with all vehicles modeled as passenger cars. The distances between adjacent vehicles ranged from 2 to 3 m, and the spacing between front and rear vehicles was 3 to 4 m, resulting in a total of 172 cars positioned within the tunnel. To ensure the accuracy of the computational results, a local grid refinement was applied at the tunnel entrances, exits, and exhaust outlets. Ren et al.^24,25^ applied Fluent software to analyze the diffusion characteristics of transient vehicle exhaust within extremely long tunnels. A two-dimensional quadrilateral structured grid was used throughout the computational domain within the tunnel. Linear grids were used to densify sensitive areas, such as the vehicle exhaust pipe and vehicle body. The total number of grid cells for both long and short vehicle distances was approximately 70,000. Based on the above results, the entire Bayi Tunnel model comprised a total of 4.26 million grid cells, as illustrated in Figs. 2 and 3.

**Fig. 4:**
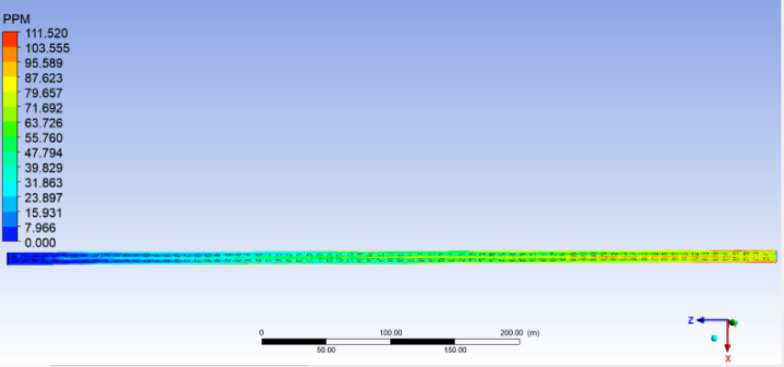
Cloud map of longitudinal CO distribution in Bayi tunnel.

**Fig. 5:**
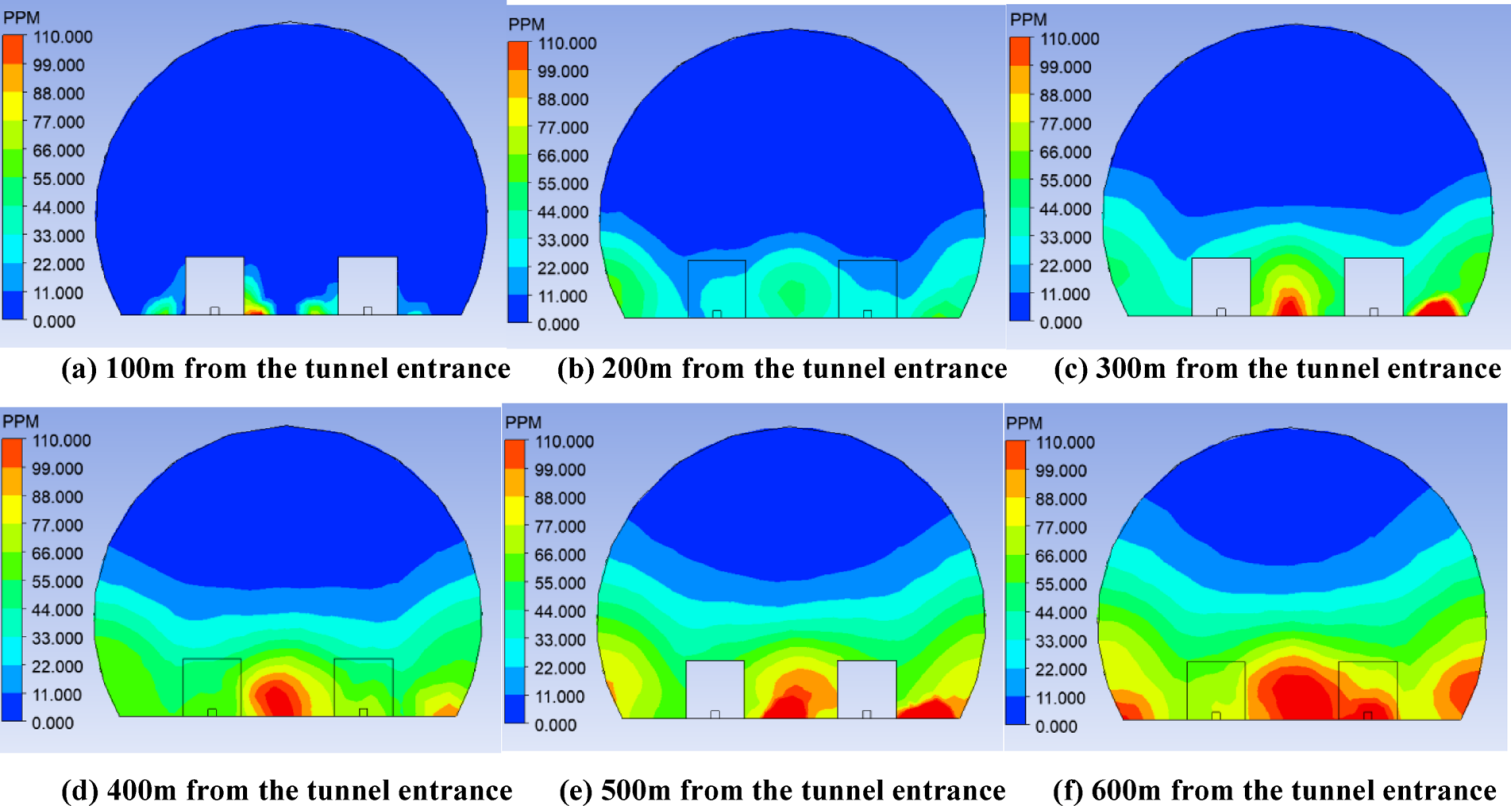
Cloud diagram of CO concentration distribution in longitudinal section of Bayi tunnel.

The computational method employed the finite volume method, with the turbulence model utilizing the k-ε model. Pressure–velocity coupling was achieved using the SIMPLEC algorithm. The results obtained from the simulation are presented in Figs. 4 and 5.

It can be seen from Fig. 4 that as the distance from the tunnel entrance increases, the CO concentration also increases, and the CO concentration in the tunnel center increases faster than that in the tunnel sides, that is, the center of the tunnel far away from the tunnel entrance is more likely to produce CO gas accumulation.

Figure 5 shows CO concentration distribution in longitudinal section of Bayi Tunnel. As the distance from the tunnel entrance increases, CO concentration gradually increases in the lower middle portion of the tunnel, while almost no CO accumulates in the upper middle portion. Furthermore, CO is primarily concentrated near the ground in the center of the tunnel. At 500 m from the tunnel entrance, CO begins to appear at the base of the tunnel arch. At 600 m from the tunnel entrance, CO concentration reaches its highest point in the lower middle portion of the tunnel.

**Fig. 6:**
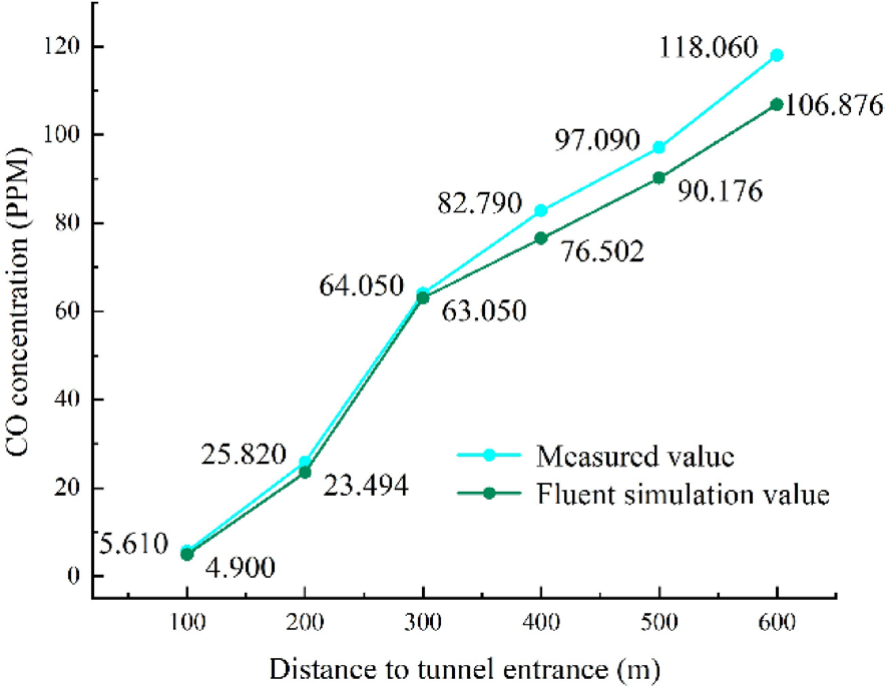
Comparison of Fluent simulated and measured values.

By comparing the Fluent simulation results with the on-site measured data from the Bayi Tunnel (see Fig. 6), it is found that the relative errors of CO concentration are generally within 10%, and the growth trends are consistent. The computational results align closely with the measured values from the tunnel. This validation of the Fluent tunnel simulation under natural ventilation conditions establishes a foundation for the study of pollutant gas concentration distribution and the optimization of ventilation methods in the Donggang Mountain secondary highway long tunnel.

### The donggang mountain secondary highway long tunnel introduction

**Fig. 7:**
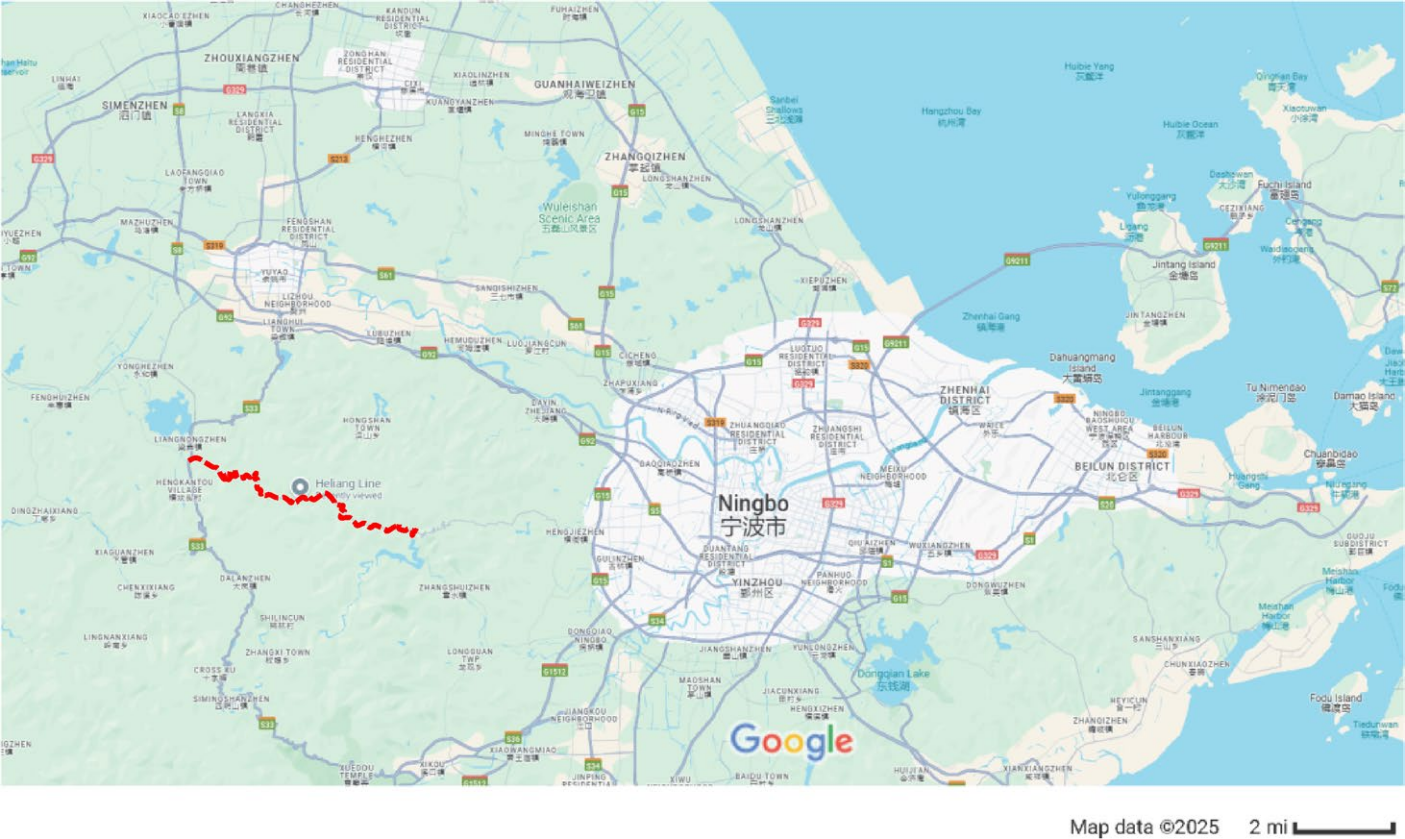
Location of Donggangshan class II extra-long highway tunnel (Google map).

**Fig. 8:**
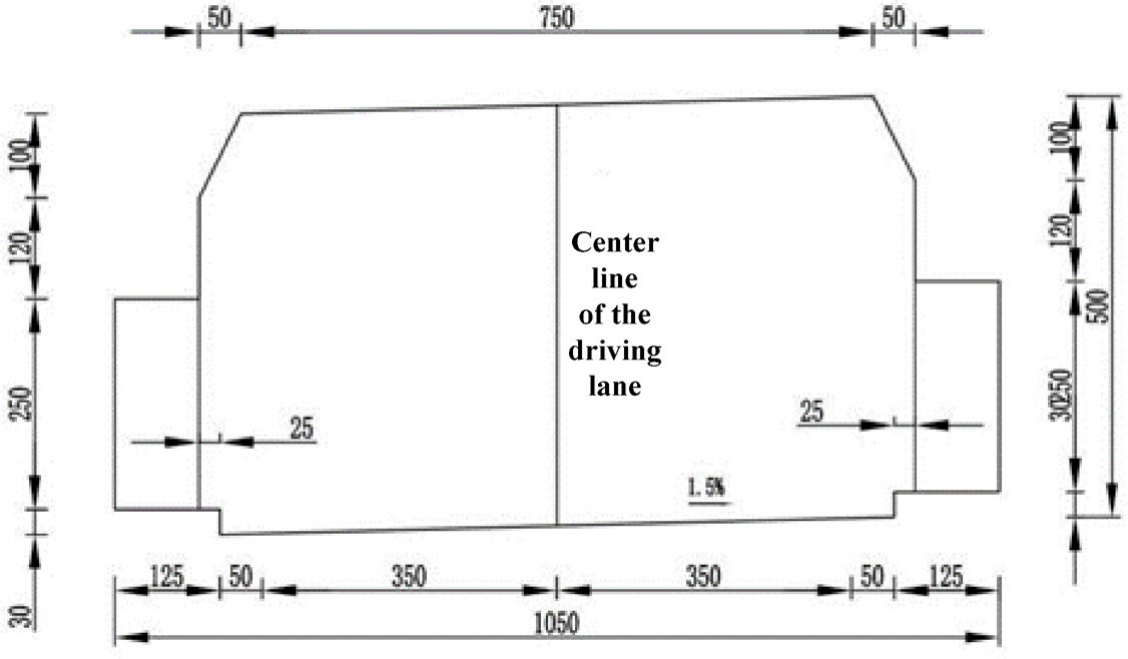
Cross-sectional dimensions of the main tunnel of the Donggangshan class II extra-long highway tunnel.

The Donggang Mountain secondary highway long tunnel is a mountain tunnel located on the Yuyiao section of Zhejiang Provincial Road S309, connecting Xiaoyun Village in Luting Township with Jinlingxia Village in Liangnong Town. The total length of the tunnel is 4045 m, and the location map is shown in Fig. 7. Figure 7 is generated by Google map (https://www.google.com/maps) and edited by the author to clarify the location of the tunnel in this paper in red line. The tunnel has a width of 10.6 m and a height of 6.7 m, with the cross-sectional dimensions of the main tunnel shown in Fig. 8.

Secondary highway tunnels, unlike fully enclosed expressway tunnels, have open entrances and exits, which means there is a possibility of pedestrians and non-motorized vehicles entering the tunnel during operation. As a result, the air quality requirements for such tunnels are higher. The Donggang Mountain secondary highway long tunnel is the main passageway for travelers from Ningbo and Shanghai to the Siming Lake scenic area, which is known for its beautiful landscapes, blending lakes and mountains, and numerous historical sites. It has become a popular tourist destination, with peak traffic flows concentrated on weekends and holidays. The tunnel experiences heavy traffic and is prone to long-distance congestion. Therefore, it is necessary to study the air quality and ventilation optimization plan under conditions of long-distance traffic congestion in the tunnel, in order to reduce the concentration of pollutant gases inside the tunnel and improve the efficiency of the ventilation.

### Donggang mountain long tunnel operational ventilation system modeling

**Fig. 9:**
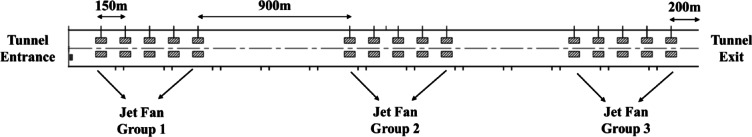
Layout of Donggangshan tunnel ventilation system.

**Fig. 10:**
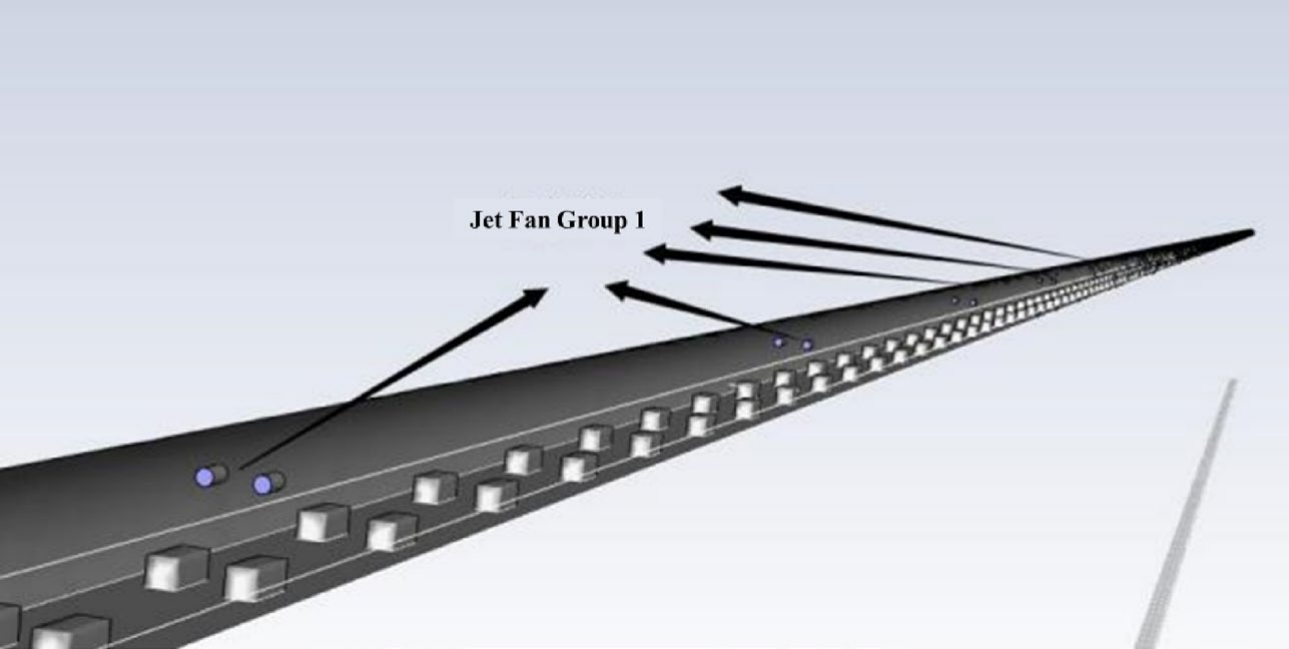
Fluent longitudinal ventilation system modelling diagrams.

The Donggang Mountain secondary highway long tunnel is located in the Siming Mountain area of Yuyao and has a subtropical monsoon climate. Meteorological data shows that the dominant wind direction throughout the year is NNE (predominantly from the northeast), with an average annual wind speed of 2.3 m/s. The wind direction corresponds to a flow from Jinlingxia Village in Luting Township towards Xiaoyun Village in Liangnong Town. Therefore, in this paper, the Fluent ventilation system model is set with Jinlingxia Village as the tunnel entrance, assuming that the driving direction aligns with the ventilation direction. The ventilation design scheme for the Donggang Mountain secondary highway long tunnel adopts a full jet longitudinal ventilation system, with three jet fan clusters arranged within the tunnel. Each jet fan cluster consists of 10 jet fans with the same power rating. Each jet fan has a power of 18.5 kW/AC380V, with an outlet wind speed of 30.7 m/s. The installation height of the fans is 5.4 m. Two fans are arranged side-by-side in each row within the fan cluster, and the distance between the side-by-side fans is 2 m. Each cluster contains five rows of fans, with a distance of 150 m between the front and rear rows. The distance from the first and third fan clusters to the tunnel entrance and exit is 200 m, while the longitudinal distance between the second fan cluster and the others is 900 m. The specific layout of the fans is shown in Fig. 9. Based on the operational characteristics of secondary highway tunnels and the potential for extreme traffic congestion, the Fluent simulation model of the longitudinal ventilation system is shown in Fig. 10.

## The impact of ventilation method on pollutant gas distribution

Under the condition of total congestion in the tunnel, the impact of different ventilation methods, including natural ventilation and natural ventilation combined with jet fans, on the distribution of pollutant gases inside the tunnel is discussed.

Due to the fact that the CO concentration setting values in the standards are based on a blockage length of 1 km, while the actual peak traffic flow and tunnel blockage length in special conditions can far exceed 1 km, this study sets the operating condition as a full-length (4 km) traffic jam scenario. The study focuses on the variation of pollutant gas concentrations in the tunnel under extreme conditions, as well as the impact of different ventilation methods on improving air quality inside the tunnel. The CO concentration below 300 ppm is selected as the benchmark for evaluating the ventilation efficiency of different ventilation schemes. The CO concentration monitoring data is taken at a height of 1.5 m from the tunnel side. Simulations are performed under different ventilation conditions (as shown in Table 1), and the resulting CO concentration data is presented in the form of curves.

**Table 1:** Different conditions with different ventilation methods.

Ventilation method	Condition	Natural wind speed (m/s)	Fan group 1	Fan group 2	Fan group 3
Natural ventilation	Condition 1	1	Off	Off	Off
	Condition 2	2			
	Condition 3	2.3			
	Condition 4	3			
	Condition 5	4.5			
Natural ventilation combined with jet fans	Condition 6	2.3	On	On	Off
	Condition 7		On	Off	On
	Condition 8		Off	On	On
	Condition9		On	On	On

### The influence of different wind speeds on natural ventilation

**Fig. 11:**
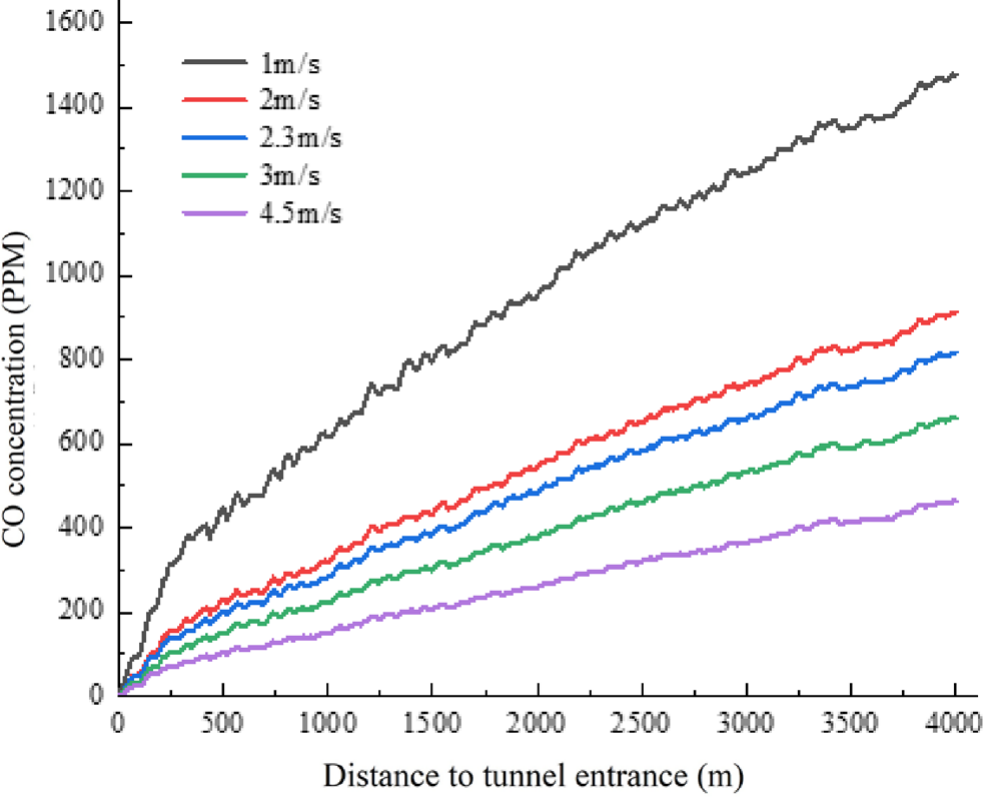
Comparison of the variation of CO concentration in the longitudinal direction of the tunnel under different wind speeds inside the tunnel.

In the case of natural ventilation, the wind speed inside the tunnel has an important impact on the distribution of CO concentration under blockage conditions. Under the different wind speeds of Operating Conditions 1–5 shown in Table 1^19,20^, the longitudinal CO concentration distribution in the tunnel is generally consistent, as shown in Fig. 11. The concentration increases linearly from the tunnel entrance to the tunnel exit and peaks at the tunnel exit. In the 0-500 m section near the tunnel entrance, the CO concentration increases rapidly, while the rate of increase slows down in the following sections. The wind speed significantly affects the peak concentration of pollutants inside the tunnel. When the wind speed inside the tunnel increases from 1 to 2 m/s, the CO concentration at the tunnel exit decreases from 1468 to 911 ppm. When the wind speed is between 2 and 4.5 m/s, for each 1 m/s increase in wind speed, the CO concentration at the tunnel exit decreases by about 200 ppm. At the same time, the wind speed also has a significant impact on the rate of increase of CO concentration. Compared with the 1 m/s and 3 m/s conditions, in the 0-500 m section near the tunnel entrance, when the wind speed increases from 1 to 3 m/s, the CO concentration growth rate in this section decreases by 62.5%. From the 500 m to the tunnel exit section, the CO concentration growth rate also decreases by 52%. Maintaining an appropriate wind speed inside the tunnel is of great significance for slowing down the growth rate of CO concentration and reducing the peak CO concentration in long highway tunnels. During traffic congestion in long highway tunnels, the ventilation system should be activated in time to ensure that the wind speed inside the tunnel is at a reasonable level, and the wind speed should not be lower than 2 m/s.

### The impact of different longitudinal jet ventilation methods

**Fig. 12:**
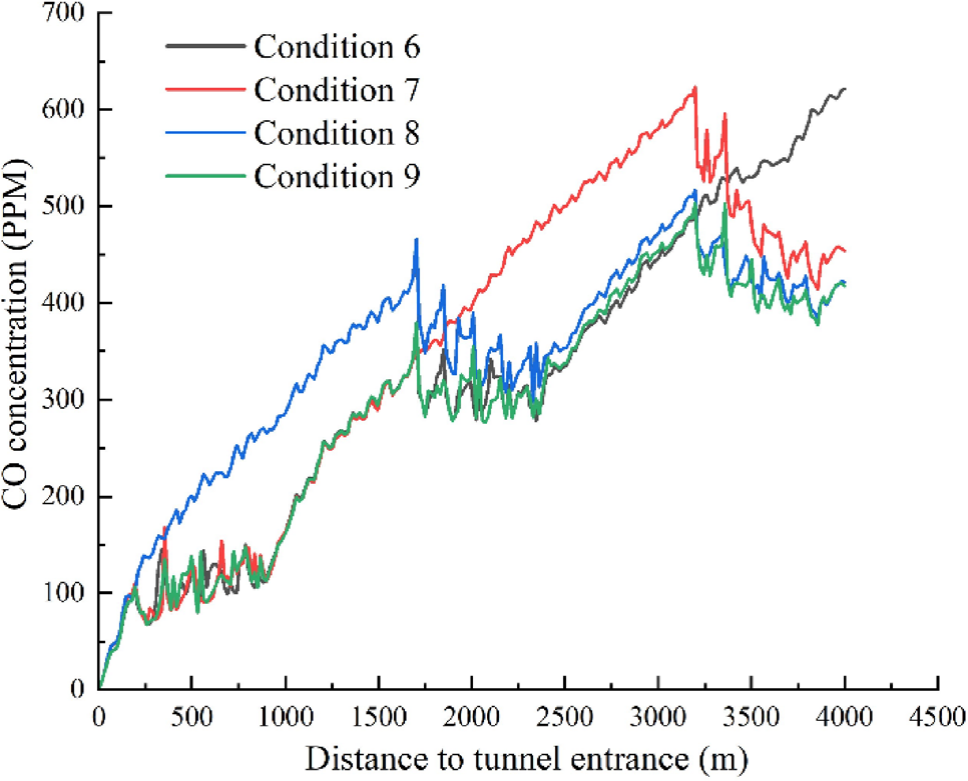
Trend of CO concentration in the longitudinal direction of the tunnel for condition 6–9.

The CO concentration curves at the 1.5-m height on the tunnel side for condition 6–9 are shown in Fig. 12. In condition 6 of Table 1, Jet Fan Groups 1 and 2 are both turned on at the design wind speed of 30 m/s, which is determined according to the survey and design data. It can be seen from Fig. 12 that Jet Fan Group 1 can maintain the CO concentration in the 200 m to 900 m section of the tunnel at around 100 ppm in condition 6. In the middle section where no fans are installed, the CO concentration increases linearly. Jet Fan Group 2 can maintain the CO concentration in the 1600 m to 2400 m section at around 300 ppm. In the section from 2500 to 4000 m of the tunnel, where no fans are turned on, the CO concentration also increases linearly, and the CO concentration at the exit exceeds 600 ppm.

In Condition 7, Jet Fan Groups 1 and 3 are both turned on at the design wind speed of 30 m/s. The longitudinal CO concentration variation curve at a height of 1.5 m in the tunnel is shown in Fig. 12. Similar to Condition 6, Jet Fan Group 1 can maintain the CO concentration in the 200 m to 900 m section of the tunnel at around 100 ppm. In the section from 1000 to 3000 m, where no fans are installed, the CO concentration increases linearly and peaks at around 600 ppm near 3100 m. In the exit section, under the influence of Jet Fan Group 3, the CO concentration decreases and is maintained at about 550 ppm.

In Condition 8, Jet Fan Groups 2 and 3 are both turned on at the design wind speed of 30 m/s. The longitudinal CO concentration variation curve at a height of 1.5 m in the tunnel is shown in Fig. 12. From the tunnel entrance to 1700 m, the CO concentration increases linearly, and in the section influenced by Jet Fan Group 2, the CO concentration is maintained at around 380 ppm. In the interval between the influence of Fan Group 2 and Fan Group 3, the CO concentration continues to increase, peaking at 590 ppm near 3200 m. In the exit section, under the influence of Jet Fan Group 3, the CO concentration remains around 550 ppm. Conditions 7 and 8 indicate that when only one of Fan Groups 1 and 2 is turned on, there is no impact on the CO concentration at the tunnel exit. Since the first group of jet fans is located 200 m from the tunnel entrance, in Condition 7, the section where the CO concentration is below 300 ppm has a length of 1500 m, while in Condition 8, the section where the CO concentration is below 300 ppm has a length of 1000 m.

By comparing Conditions 6, 7, and 8, it can be observed that in the case of total congestion in the tunnel, opening more fans near the tunnel entrance is more effective in reducing the overall CO concentration within the tunnel.

**Fig. 13:**
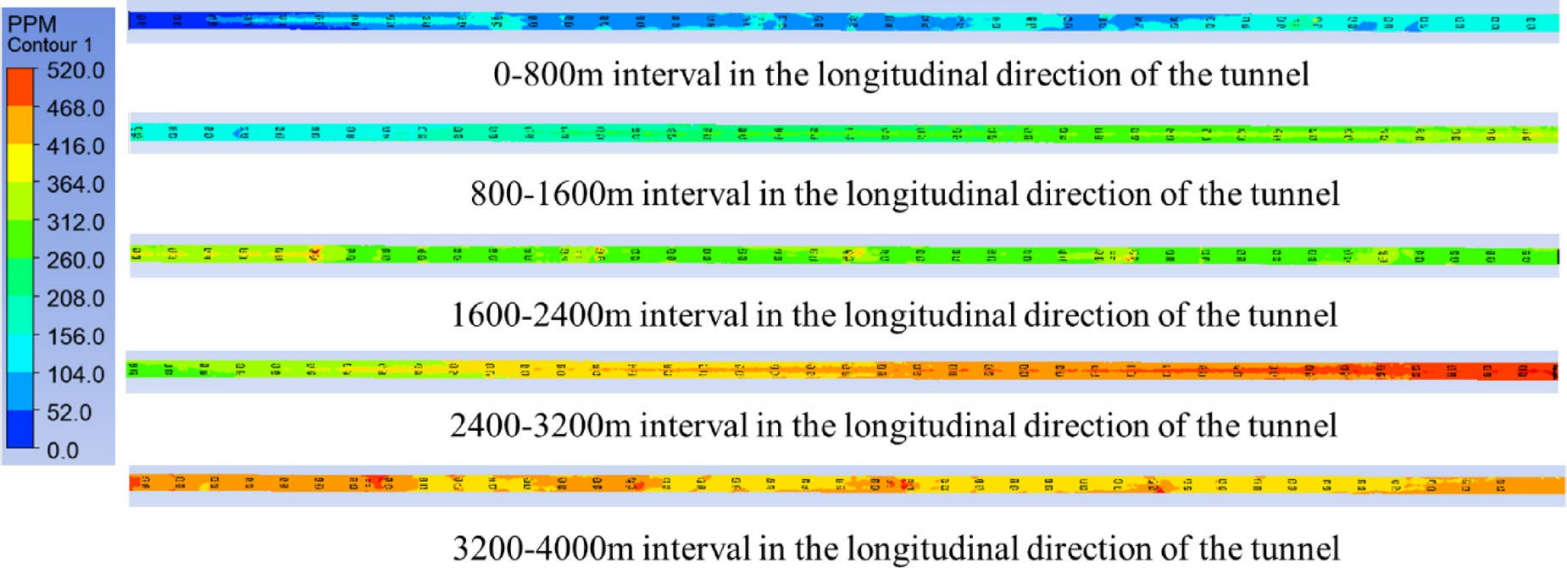
Variation of CO concentration in the longitudinal direction of the tunnel for condition 9.

Condition 9 involves all three jet fan groups being turned on at the design wind speed of 30 m/s. The longitudinal CO concentration variation curve at a height of 1.5 m in the tunnel is shown in Figs. 12 and 13. It can be seen that the CO concentration distribution along the tunnel exhibits three distinct plateau regions in the sections where the fan groups are located. In the section influenced by Jet Fan Group 1, the CO concentration fluctuates around 100 ppm, in the section influenced by Jet Fan Group 2, the CO concentration fluctuates around 300 ppm, and in the section influenced by Jet Fan Group 3, the CO concentration stabilizes around 400–430 ppm. Compared to turning on two fan groups, the CO concentration at the tunnel exit can be reduced by 100 ppm.

**Fig. 14:**
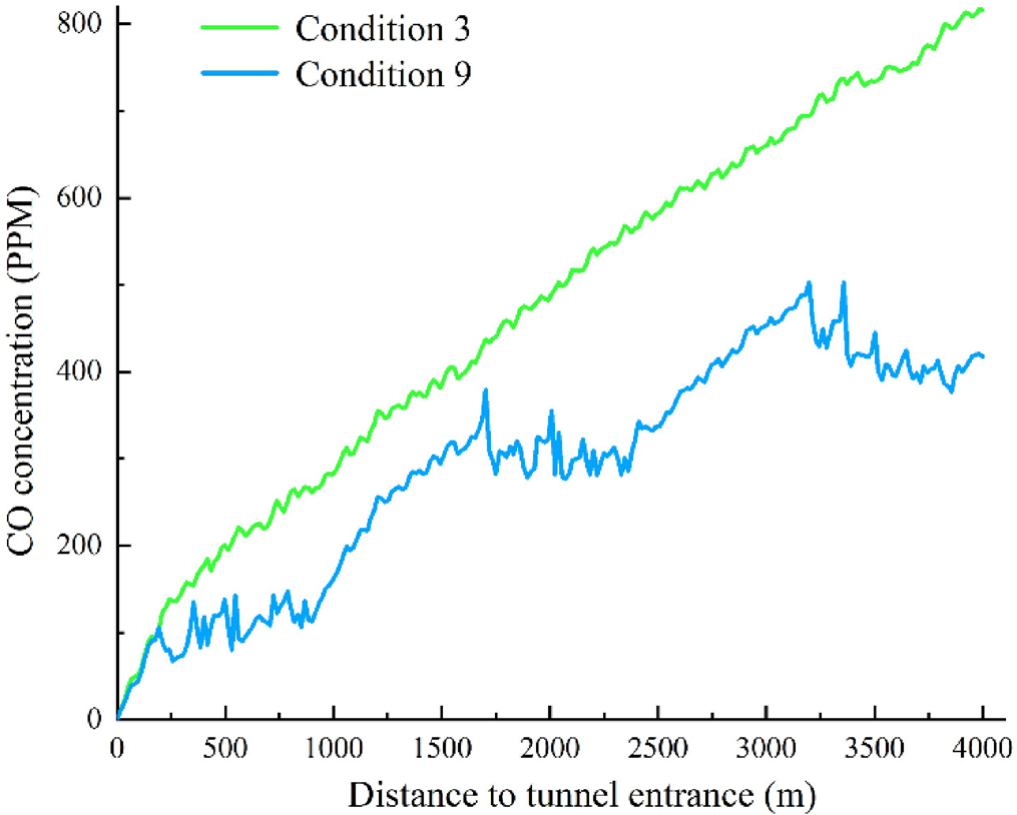
Comparison of changes in CO concentration between natural ventilation and full operation of the fan set.

Through a comparison between natural ventilation and full operation of the fan groups (as shown in Fig. 14), when the tunnel is fully blocked, relying solely on natural ventilation will result in the CO concentration at the tunnel exit exceeding 800 ppm, which significantly surpasses the allowable value in the standards. When all three jet fan groups are turned on, the CO concentration at the tunnel exit is reduced to approximately 400 ppm, and the peak CO concentration decreases by 50%. At the same time, the section of the tunnel where the CO concentration remains below 300 ppm extends for 2500 m. In the sections where the jet fan groups are located, a CO concentration “platform area” of 800–1000 m in length appears, where the CO concentration remains relatively stable, effectively slowing the rate of CO concentration increase and reducing the peak value within the tunnel.

## Optimization of combined ventilation method

From the analysis in the previous section, it is evident that the primary function of jet fans is to accelerate the airflow inside the tunnel, maintaining a relatively stable CO concentration in the section where the fan groups are located. However, jet fans alone cannot directly reduce the CO concentration within the entire tunnel. On the other hand, ventilation shafts can introduce fresh outside air^38,39^, diluting and neutralizing the internal pollutants, which helps to lower the average concentration of air pollutants in long tunnels.

### Natural ventilation and shaft ventilation combination

In principle, a ventilation shaft can be set up for long tunnels, but due to the limited research and application on the combined effect of ventilation shafts and jet fan groups, the more common ventilation design scheme currently is full-jet longitudinal ventilation. In fact, although constructing a ventilation shaft increases the initial construction costs, it has significant long-term economic and social benefits^32,33^. The construction of a shaft can not only reduce the installation costs of fans but also lower the operational costs of fans during the tunnel’s operation.

**Fig. 15:**
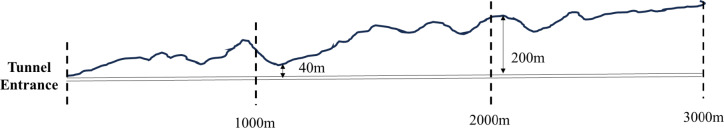
Topographic profile of the Donggangshan secondary highway super-long tunnel.

According to the geological survey profile of the Donggangshan Long Highway Tunnel (shown in Fig. 15), the distance from the tunnel entrance to the mountaintop ranges from 40 to 200 m, making it suitable for the installation of a ventilation shaft.

**Fig. 16:**
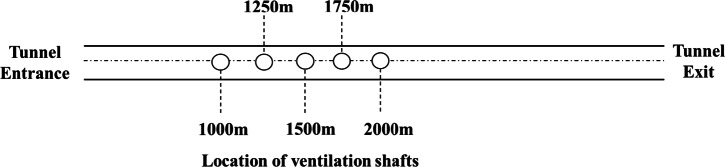
Ventilation shaft alternative location plan.

According to relevant tunnel engineering cases on the installation of ventilation shafts^39,40^, the air supply velocity for the tunnel shaft can range from 10 to 18 m/s. To reduce the civil engineering workload of the shaft, a shaft radius of 2 m is selected, with a designed air supply volume of 126 m^3^/s and a wind speed of 10 m/s. Ventilation effect simulations are carried out at five locations, spaced 250 m apart, from 1000 to 2000 m from the tunnel entrance, as shown in Fig. 16.

**Fig. 17:**
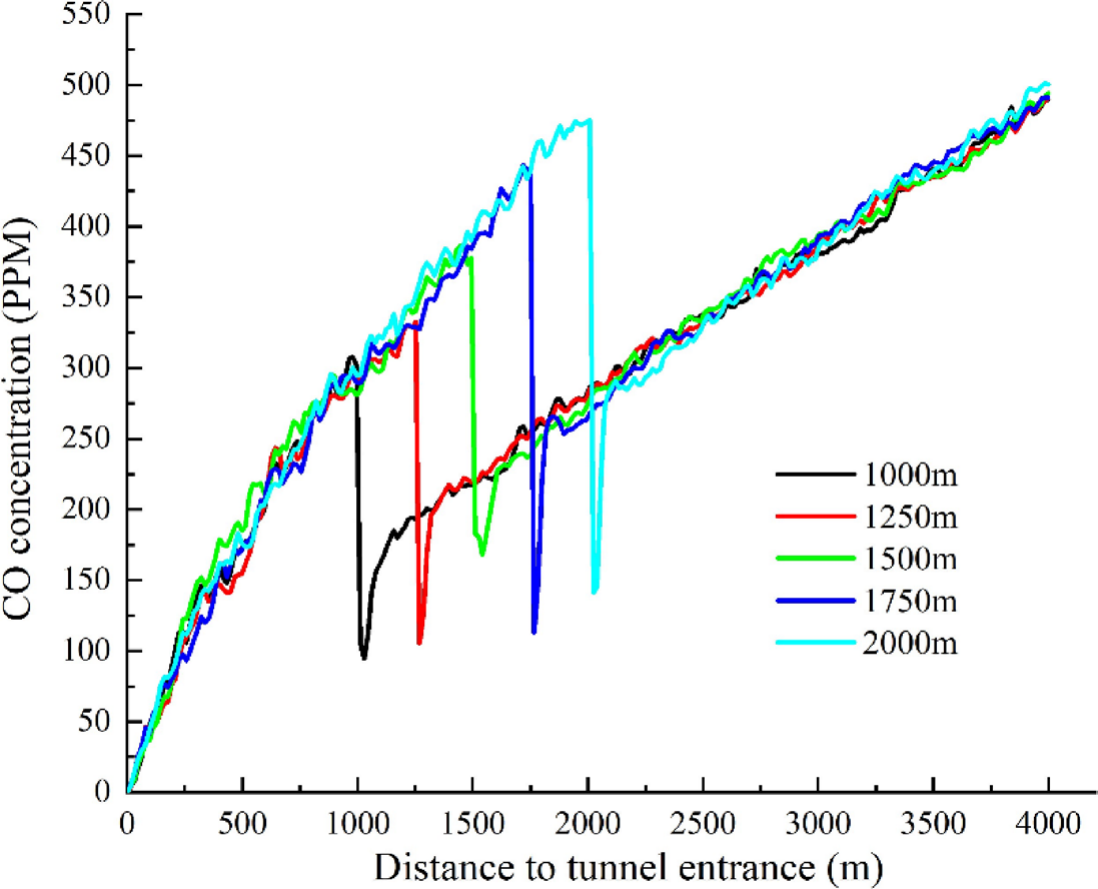
Comparison of ventilation efficiency at different distances from ventilation shaft to tunnel entrance.

The analysis results are shown in Fig. 17. When the ventilation shaft is in operation, it can reduce the CO concentration at its location by approximately 200–350 ppm, and lower the CO concentration in the 1000–1500 m downstream of the shaft compared to the concentration in front of the shaft. The closer the ventilation shaft is to the tunnel entrance, the lower the average CO concentration inside the tunnel after the reduction.

**Fig. 18:**
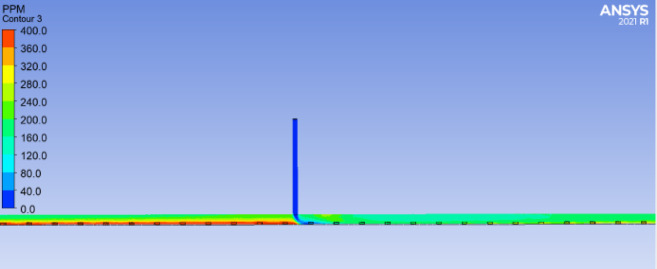
Profiles of CO concentration changes before and after ventilation shafts.

**Fig. 19:**
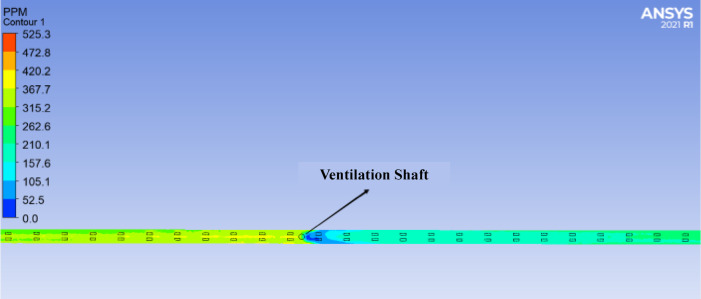
Top view of the change in CO concentration before and after the ventilation shafts.

Based on the terrain conditions of the Donggangshan Long Tunnel and the safety CO concentration standards for humans, it is more reasonable to place the ventilation shaft at 1250 m from the tunnel entrance. As shown in Figs. 18 and 19, before the ventilation shaft at 1250 m, the CO concentration increases continuously along the tunnel. At the ventilation shaft, the concentration decreases sharply, and the growth rate of CO concentration slows down after the shaft. However, since the longitudinal distance of the long highway tunnel exceeds 3000 m, the CO concentration in the middle and rear sections of the tunnel will again rise to higher values. Therefore, it is necessary to combine the use of jet fans to reduce the overall CO concentration inside the tunnel.

### Natural ventilation, jet fans, and shaft combined ventilation

According to the previous analysis, setting the shaft at a distance of 1250 m from the tunnel entrance is considered reasonable. Next, we further explore the ventilation effect of the combined system of natural ventilation + jet fans + shaft, simulating conditions 10–12 as listed in Table 2, and discussing the optimization of the combined ventilation scheme.

**Table 2:** Different working conditions of natural ventilation, jet fan and shaft combined ventilation.

Ventilation methods	Condition	Natural wind speed (m/s)	Fan group 1	Fan group 2	Fan group 3	Ventilation shaft
Natural Ventilation, Jet Fans, and Ventilation Shaft	Condition 10	2.3	Off	Off	On	On
	Condition 11		Off	On	On	
	Condition 12		On	On	On	

**Fig. 20:**
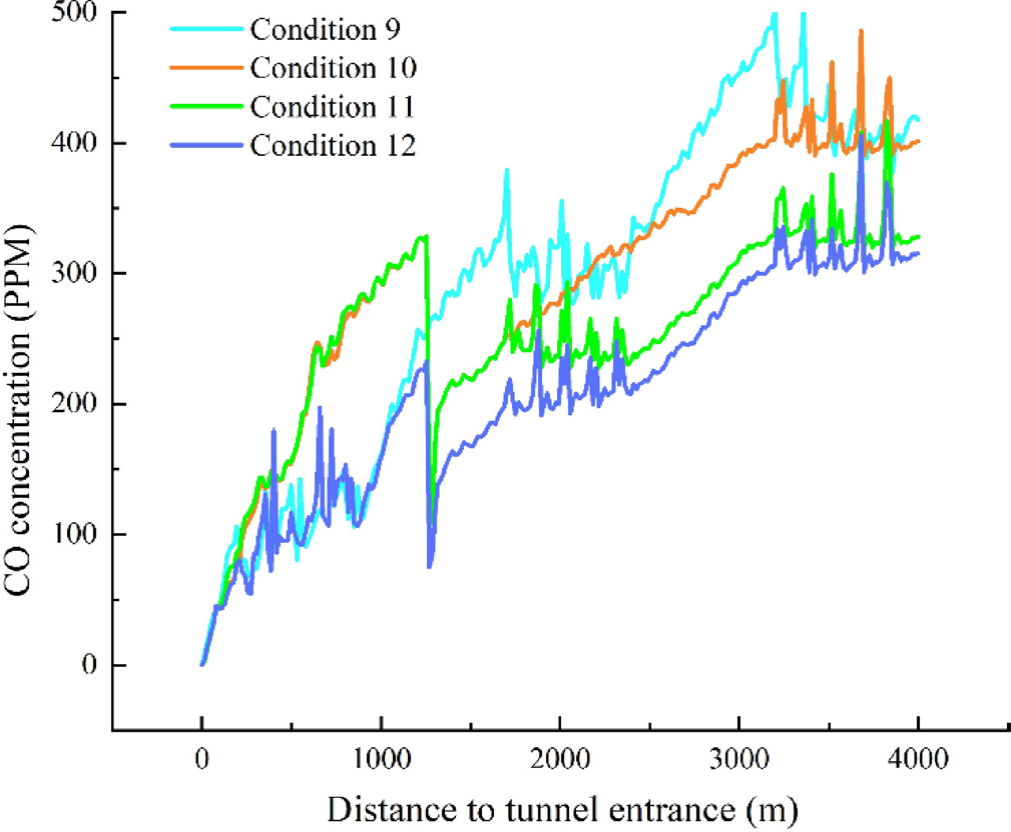
Variation curve of CO concentration under different ventilation methods.

Figure 20 shows a comparison of the simulation results for different working conditions. A comparative analysis between working conditions 10 and 9 reveals that, from the tunnel entrance to the ventilation shaft location, CO concentration in working condition 10 continues to increase, reaching 320 ppm. In contrast, in working condition 9, due to the action of the first jet fan group, the CO concentration increases more slowly in the 200–800 m section of the tunnel, then increases at a faster rate between 800 and 1250 m, reaching 250 ppm. After the ventilation shaft is turned on, in working condition 10, the CO concentration drops sharply to 100 ppm at the shaft, lower than the CO concentration in working condition 9 at that location. Due to the action of the second jet fan group, the CO concentration in working condition 9 again experiences a slower increase along the tunnel length. At this point, the CO concentrations of the two schemes are similar, but subsequently, the increase rate of CO concentration in working condition 9 is faster than that in working condition 10. At the tunnel exit, the CO concentration in working condition 10 is about 50 ppm lower than that in working condition 9. By observing the CO concentration variation along the tunnel length in working condition 9, it can be seen that without turning on the ventilation shaft, the rate of increase in CO concentration before and after the jet fans is similar. However, when the ventilation shaft is not turned on, the introduction of fresh air from outside into the tunnel through the shaft results in a 50% reduction in the rate of increase in CO concentration after the shaft compared to the full jet fan operation.

## Conclusion

This paper uses the Donggangshan secondary highway long tunnel as the engineering background and employs fluid dynamics software Fluent to investigate the impact of different ventilation methods on the distribution characteristics of pollutants and their ventilation effects during extreme traffic congestion. The main conclusions are summarized as follows:

1. Under the predominant annual wind direction, pollutant concentrations in long highway tunnels increase linearly during prolonged traffic congestion, peaking near the tunnel exit. Maintaining a tunnel wind speed of at least 2 m/s is critical to slow pollutant growth and lower peak concentrations.
2. Activating jet fan groups helps maintain a relatively stable pollutant level within a 600–800 m section around the fans. Starting fan groups closer to the tunnel entrance is more effective in reducing the overall CO concentration during extended congestion. When all three jet fan groups are activated, the CO concentration at the tunnel exit decreases by approximately 400 ppm, with a 50% reduction in peak CO levels.
3. The addition of a ventilation shaft at 1250 m from the tunnel entrance leads to a sharp drop in CO concentration at that location. Introducing fresh air through the shaft reduces the CO growth rate downstream by 50% compared to jet fan ventilation alone.
4. Considering both ventilation effectiveness and operational costs, the combined use of the ventilation shaft with the second and third jet fan groups is identified as the optimal ventilation strategy for the Donggangshan Tunnel, contributing to greener and more energy-efficient operation.

It should be noted that this study has certain limitations. Firstly, the investigation of pollutants is limited to CO, without considering the distribution and impacts of other vehicular emissions such as nitrogen oxides or particulate matter. Secondly, the model does not account for variations in vehicle types (e.g., cars, trucks), which may lead to differences in actual emission characteristics and ventilation demands. Additionally, this research does not incorporate extreme weather conditions (e.g., strong crosswinds, heavy rain) or emergency scenarios such as tunnel fires, which could significantly alter pollutant dispersion patterns and ventilation system performance. These limitations could affect the generalizability of the findings and should be addressed in future research.

For future tunnel ventilation optimization, research can be expanded to include the upgrading of ventilation facilities and the optimization of traditional ventilation control systems. New tunnel ventilation control systems should be based on feedback data from gas pollutant monitoring systems to adjust the operation of the fans, ensuring that the air quality inside the tunnel meets driving requirements. Depending on feedback signal timing, highway tunnel ventilation control modes can be roughly divided into feedforward, feedback, and real-time feedback systems. However, due to the strong time-varying, delayed, and nonlinear characteristics of highway ventilation systems, traditional control systems cannot comprehensively account for all factors and establish accurate mathematical models. Fuzzy control theory, genetic algorithms, neural networks, and other methods can be employed to optimize traditional ventilation control systems, making them more adaptive, real-time adjustable, energy-efficient, and capable of meeting the ventilation needs during tunnel congestion, while saving energy during normal operation and preventing fan damage from idling.

## Data Availability

The datasets used and analyzed during the current study available from the corresponding author on reasonable request.
